# Burden and attitude to resistant and refractory migraine: a survey from the European Headache Federation with the endorsement of the European Migraine & Headache Alliance

**DOI:** 10.1186/s10194-021-01252-4

**Published:** 2021-05-18

**Authors:** Simona Sacco, Christian Lampl, Antoinette Maassen van den Brink, Valeria Caponnetto, Mark Braschinsky, Anne Ducros, Patrick Little, Patricia Pozo-Rosich, Uwe Reuter, Elena Ruiz de la Torre, Margarita Sanchez Del Rio, Alexandra J. Sinclair, Paolo Martelletti, Zaza Katsarava, On behalf of the Burden and Attitude to Resistant and Refractory (BARR) Study Group, Genci Cakciri, Pavllo Djamandi, Serla Grabova, Gleni Halili, Jera Kruja, Altin Kuqo, Diana Naco, Aida Quka, Ladjola Stefanidhi, Gentian Vyshka, Ilirjana Zekja, Osvaldo Bruera, Dilver Gómez, Brenda Guitian, Juan Carlos Roma, Ike Leon Chen, Christian Lampl, Samira Bashirova, Maxim Linkov, Daan Van Den Abbeele, Geraldine Vanderschueren, Roberta Araujo, Renato Arruda, Antônio Catharino, Jovana Ciriaco, Amauri Dalla Corte, Ricardo Dornas, Bernardo Felsenfeld, André Fonseca Taufner, Yara Fragoso, Rubens Hurtado, Daniel Isoni Martins, Renata Londero, Luciano Melo, Kelton Stivenson Mignoni, Paulo Victor Sgobbi De Souza, Marcio Nattan Souza, Sevdzhan Osman, Veronika Baltzer, Luis Fabian Pacheco Mosquera, Ivan Dubroja, Zlatko Hucika, Marijana Lisak, Arijana Lovrencic-Huzjan, Ivo Lušić, Darija Mahovic Lakusic, Petr Mikulenka, Pavel Řehulka, Faisal Mohammad Amin, Sonja Antic, Zainab Fakhril-Din, Jakob Moeller-Hansen, Signe Munksgaard, Ana Maria Nan, Lanfranco Pellesi, Henrik Schytz, Manuel Vides, Kati Braschinsky, Mark Braschinsky, Ülle Krikmann, Anne Ducros, Caroline Roos, Alexandre Cauchie, Lucas Christian, Evelyne Guégan-Massardier, Genevieve Demarquay, Geraud Gilles, Jerome Mawet, Emmanuelle Kuhn, Michel Lanteri Minet, Mihaela Bustuchina Vlaicu, Xavier Moisset, Mirela Muresan, Mitra Najjar-Ravan, Pierric Giraud, Sabine Simonin, Solène De Gaalon, George Chakhava, Miranda Demuria, George Gegelashvili, Nana Kapanadze, Angela Antonakakis, Charly Gaul, Stefanie Förderreuther, Jana-Isabel Huhn, Sardor Ibragimov, Katharina Kamm, Bianca Raffaelli, Reiner Czaniera, Ruth Ruscheweyh, Uwe Reuter, Evangelia Gavanozi, Georgios Karagiorgis, Theodoros Mavridism, Csaba Ertsey, Dubey Shubham, Antonia Dewi Callista Tanowi, Stefanus Erdana Putra, Deby Wahyuning Hadi, Leny Kurnia, Musadir Nasrul, Maria Albanese, Fabio Antonaci, Gian Maria Asioli, Roberta Baschi, Enrico Bentivegna, Nicoletta Brunelli, Salvatore Caratozzolo, Teresa Catarci, Alessandra Cherchi, Ilenia Corbelli, Alfredo Costa, Ciro De Luca, Alberto Doretti, Valentina Favoni, Natascia Ghiotto, Maria Adele Giamberardino, Luca Giani, Giorgio Zanchin, Flora Govone, Giovanni Grillo, Edoardo Mampreso, Paolo Martelletti, Andrea Negro, Raffaele Ornello, Marcello Pasculli, Umberto Pensato, Maria Pia Addolorata Prudenzano, Simone Quintana, Rosaria Rapisarda, Michele Romoli, Antonio Russo, Marco Russo, Simona Sacco, Valerio Spuntarelli, Cindy Tiseo, Angelo Torrente, Alessandro Vacca, Giovanna Vaula, Alessandro Viganò, Simone Vigneri, Aija Freimane, Evita Slosberga, Linda Zvaune, Hui Jan Tan, Cherilyn Fenech, Rafael Cobilt-Catana, Yazmín De La Garza Neme, Marco Martínez, Jefferson Voltaire Proaño Narvaez, Andrea Rodriguez Herrera, Damaris Vazquez, Oxana Grosu, Adi Jakupi, Espen Saxhaug Kristoffersen, Erling Tronvik, Bendik S. Winsvold, Mehboob Azhar, Maria Teresa Reyes Alvarez, Leidi Vilchez Fernandez, Greg David Dayrit, Ewa Katarzyna Czapinska-Ciepiela, Michal Fila, Anna Gryglas-Dworak, Marina Couto, Paula Esperança, Axel Ferreira, Raquel Gil-Gouveia, Ana Goncalves, Margarida Lopes, Miguel Lourenço, Jorge Machado, Manuel Marinho, Miguel Angelo Miranda, Filipe Palavra, Elsa Parreira, Isabel Pavao Martins, Liliana Pereira, José Maria Pereira Monteiro, Pavel Leahu, Stefan Aloman, Ekaterina Abramova, Leila Akhmadeeva, Anastasia Belopasova, Inna Bogdanova, Mariya Chernyak, Miss Epifanova, Elena Fedorova, Anton Felbush, Maria Karpova, Daria Korobkova, Daria Korotkova, Nina Latysheva, Tatiana Makeeva, Kristina Mikhalkina, Vera Osipova, Olga Roshchina, Anna Serga, O. V. Serousova, Yulia Sidorova, Iaroslav Skiba, Kirill Skorobogatykh, Nina Vashchenko, Slobodan Apostolski, Nevenka Buder, Aleksandar Kopitovic, Jovanovic Mirjana, Ana Podgorac, Dejan Rakic, Svetlana Simić, Martinovic Zarko, Jasna Zidverc Trajkovic, Isabel Beltran-Blasco, María Dolores Calabria Gallego, Samuel Díaz Insa, David Ezpeleta, María Fernández, David García-Azorín, Nuria González-García, Angel L. Guerrero, Edelmira Guillamon, Jaime Herreros Rodriguez, Almudena Layos-Romero, Vicente Medrano, Ane Mínguez-Olaondo, Santiago Navarro Muñoz, Martí Paré Curell, Patricia Pozo-Rosich, Marta Ruibal, Jose Maria Sanchez Alvarez, Margarita Sanchez Del Rio, Sonia Santos, Rafael Soler, Javier Viguera, Ramon Zabalza, Tagwa Abdelrahman, Salih Boushra Abobaker Hamza, Mohammed Nasir Mustafa, Lars Edvinsson, Andreas Gantenbein, Isabella Maraffi, Emile Couturier, Thijs Dirkx, Michel Hoebert, Wpj Van Oosterhout, Mulleners Wim, Rachel Zwartbol, Mesut Bakır, Hatice Demirel, Ali Kemal Erdemoglu, Devrimsel H. Ertem, Sinan Gönüllü, Elif Ilgaz Aydinlar, Levent Ertuğrul Inan, Berkay Ölmez, Taner Ozbenli, Aynur Özge, Derya Uluduz, Tugba Uyar Cankay, Pinar Yalinay Dikmen, Amrit Bhushan Saxena, Myroslav Bozhenko, Nataliya Bozhenko, Rostyslav Bubnov, Olena Tsurkalenko, Ishaq Abu-Arafeh, Luis Idrovo, Sarah Miller, Niranjanan Nirmalananthan, Alexandra Sinclair, Esteban Taleti, Alex Valori, William Whitehouse, Adam Zermansky, Myat Thura

**Affiliations:** 1 Neuroscience section – Department of Biotechnological and Applied Clinical Sciences and (Edificio Coppito 2), University of L’Aquila, Via Vetoio, 67100 L’Aquila, Italy; 2 Regional Referral Headache Center of the Abruzzo Region, ASL Avezzano-Sulmona-L’Aquila, L’Aquila, Italy; 3 Department of Neurology, Headache Medical Centre Linz, Hospital Barmherzige Brüder, Centre of Integrative Medicine (ZiAM) Ordensklinikum Linz, Linz, Austria; 4 Division of Pharmacology, Department of Internal Medicine, Erasmus MC University Medical Center Rotterdam, Rotterdam, The Netherlands; 5 Headache Clinic, Department of Neurology, Tartu University Clinics, Tartu, Estonia; 6 Headache Unit, Neurology Department, Montpellier University Hospital and Montpellier University, Montpellier, France; 7 European Migraine & Headache Alliance (EMHA), Brussels, Belgium; 8 Headache Unit, Neurology Department, Vall d’Hebron University Hospital, Barcelona, Spain; 9 Headache and Neurological Pain Research Group, Department de Medicina, Universitat Autònoma de Barcelona, Vall d’Hebron Research Institute, Barcelona, Spain; 10 Charité Universitätsmedizin Berlin, Department of Neurology, Charité Universitätsmedizin Berlin, Berlin, Germany; 11 Neurology Department, Clinica Universidad de Navarra, Madrid, Spain; 12 Metabolic Neurology, Institute of Metabolism and Systems Research, College of Medical and Dental Sciences, University of Birmingham, Birmingham, UK; 13 Centre for Endocrinology, Diabetes and Metabolism, Birmingham Health Partners, Birmingham, UK; 14 Department of Clinical and Molecular Medicine, Sapienza University of Rome, Rome, Italy; 15 Regional Referral Headache Center of the Lazio Region, Sant’Andrea Hospital, Rome, Italy; 16 Christian Hospital, Unna, Germany; 17 Department of Neurology, University of Duisburg-Essen, Essen, Germany; 18 EVEX Medical Corporation, Tbilisi, Georgia; 19 IM Sechenov First Moscow State Medical University (Sechenov University), Moscow, Russian Federation

**Keywords:** Migraine, Resistant migraine, Refractory migraine, Migraine care

## Abstract

**Background:**

New treatments are currently offering new opportunities and challenges in clinical management and research in the migraine field. There is the need of homogenous criteria to identify candidates for treatment escalation as well as of reliable criteria to identify refractoriness to treatment. To overcome those issues, the European Headache Federation (EHF) issued a Consensus document to propose criteria to approach difficult-to-treat migraine patients in a standardized way. The Consensus proposed well-defined criteria for resistant migraine (i.e., patients who do not respond to some treatment but who have residual therapeutic opportunities) and refractory migraine (i.e., patients who still have debilitating migraine despite maximal treatment efforts).

The aim of this study was to better understand the perceived impact of resistant and refractory migraine and the attitude of physicians involved in migraine care toward those conditions.

**Methods:**

We conducted a web-questionnaire-based cross-sectional international study involving physicians with interest in headache care.

**Results:**

There were 277 questionnaires available for analysis. A relevant proportion of participants reported that patients with resistant and refractory migraine were frequently seen in their clinical practice (49.5% for resistant and 28.9% for refractory migraine); percentages were higher when considering only those working in specialized headache centers (75% and 46% respectively). However, many physicians reported low or moderate confidence in managing resistant (8.1% and 43.3%, respectively) and refractory (20.7% and 48.4%, respectively) migraine patients; confidence in treating resistant and refractory migraine patients was different according to the level of care and to the number of patients visited per week. Patients with resistant and refractory migraine were infrequently referred to more specialized centers (12% and 19%, respectively); also in this case, figures were different according to the level of care.

**Conclusions:**

This report highlights the clinical relevance of difficult-to-treat migraine and the presence of unmet needs in this field. There is the need of more evidence regarding the management of those patients and clear guidance referring to the organization of care and available opportunities.

**Supplementary Information:**

The online version contains supplementary material available at 10.1186/s10194-021-01252-4.

## Background

New migraine treatments, both acute and preventative, such as lasmiditan, monoclonal antibodies (mAbs) targeting the calcitonin gene-related peptide (CGRP) pathway, and gepants are changing the landscape of migraine treatment offering new opportunities and challenges [1–5]. Those treatments raised the issue of appropriate selection of patients because their direct cost is higher than the conventional medications [6]. Additionally, despite having a high responder rate, a proportion of patients still do not respond according to conventionally accepted definitions [7–10]. A recent real-life observational study found that 38% of patients who failed all available preventatives were non-responders after 6 months of treatment to one CGRP targeting monoclonal antibody (erenumab) [11].

In order to move forward in the field of difficult-to-treat migraine patients, the European Headache Federation (EHF) with the endorsement of the European Migraine & Headache Alliance (EMHA) issued a Consensus document to propose criteria to approach those patients [12]. In details, the new definitions of the difficult to treat migraine included non-response to acute and preventative medications. So, two diagnostic categories were identified, i.e., resistant and refractory migraine. First, 8 days with debilitating migraine despite intake acute antimigraine medication was defined as the threshold. Further, patients who tried three different classes of migraine preventative and still suffer eight debilitating migraine days classify for resistant migraine. In order to be defined as refractory, failure to all available classes of migraine preventatives, including mAbs targeting the CGRP pathway, is required.

Therefore, the aim of this study was to better understand the clinical reality and the attitude of physicians involved in migraine care toward these patients.

## Methods

### Study design, setting, and participants

We conducted a web-questionnaire-based cross-sectional international study involving headache physicians. All physicians involved in the care of patients with headache, without any restriction referring to country of residency, specialization, and years of experience in headache care were entitled to fill the questionnaire.

The e-questionnaire was shared at the 2020 annual EHF virtual conference and by advertisement on social media. Additionally, the questionnaire was publicized by mailing lists and national websites by the national headache societies affiliated to the EHF. Sharing was started in July 2020 and the data base was locked on 03 October 2020.

### Instruments and data collection

The e-questionnaire was developed by discussion and consensus within the study panel (Supplement 1). The questionnaire was built online using Redcap®, a software for designing research databases [13].

Before starting to fill-out the questionnaire participants were provided with a figure summarizing the definitions of resistant and refractory migraine and with the link to the published Consensus article [12].

Each participant had to report gender, specialization, years of experience in headache medicine, the work setting and the number of patients visited per week.

Work settings categories were defined, according to EHF definitions of the level of care [14], as follows: 1) first level of care - General primary care defined as first-line headache service (accessible first contact for most people with headache); 2) second level of care - Special-interest headache care defined as ambulatory care delivered by physicians with a special interest in headache; 3) third level of care - Headache Specialists Centers defined as advanced multidisciplinary care delivered by headache specialists in hospital-based centers. These definitions [14] were also provided as a part of the e-questionnaire.

### Data protection, sharing, and ethics

The questionnaire was not anonymous, and participants were requested to provide consent to be listed as contributors to the study. Guarantee was provided referring to the use of the provided information only in aggregated forms and not at an individual level. Group authorship was granted to all those completing the questionnaire (Burden and Attitude to Resistant and Refractory [BARR] Study Group).

As the study did not involve use data from single patients, we did not ask for an Ethic Committee approval. The data base of this study is stored at the University of L’Aquila and is available upon reasonable request form authorities and researchers by contacting the corresponding author.

### Statistical analyses

We used descriptive statistics and provided distributions of the responses to selected questions. Chi-squared test was performed to compare frequencies among variables of interest. The level of significance was set at *P* < 0.05. All statistical analyses were performed with SPSS Statistics 21.0.

## Results

**Fig. 1:**
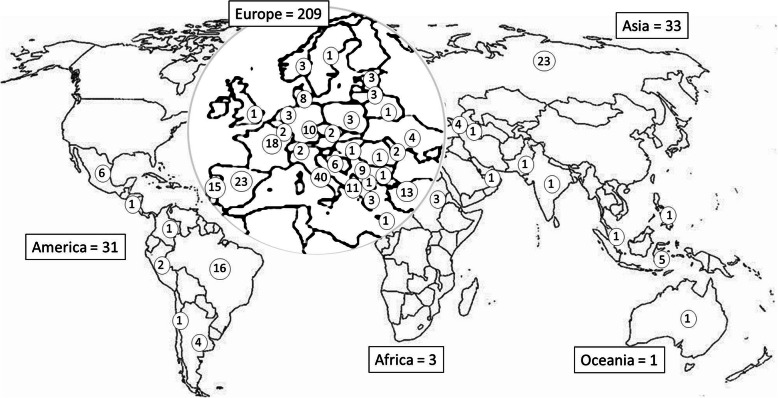
Distribution by country of participating physicians

The present paper is based on questionnaires filled by 277 physicians (*n* = 133; 48.0% female); 15 questionnaires were discarded because they were incomplete. Distribution by country is reported in Fig. 1. Characteristics of participants according to years of practice in headache medicine, specialty, and work setting are reported in Table 1.

**Table 1:** Characteristics of the participants who completed the survey

Characteristics	n	%
Females	133	48.0
Years in headache medicine after graduation		
≤ 5 years	73	26.4
6–10 years	54	19.5
> 10	150	54.2
Specialty		
Neurology	250	90.3
Other	27	9.7
Work setting		
General primary care/general neurology	83	30.0
Special interest headache care	71	25.6
Headache specialist center	123	44.4
Academic center	131	47.3

**Fig. 2:**
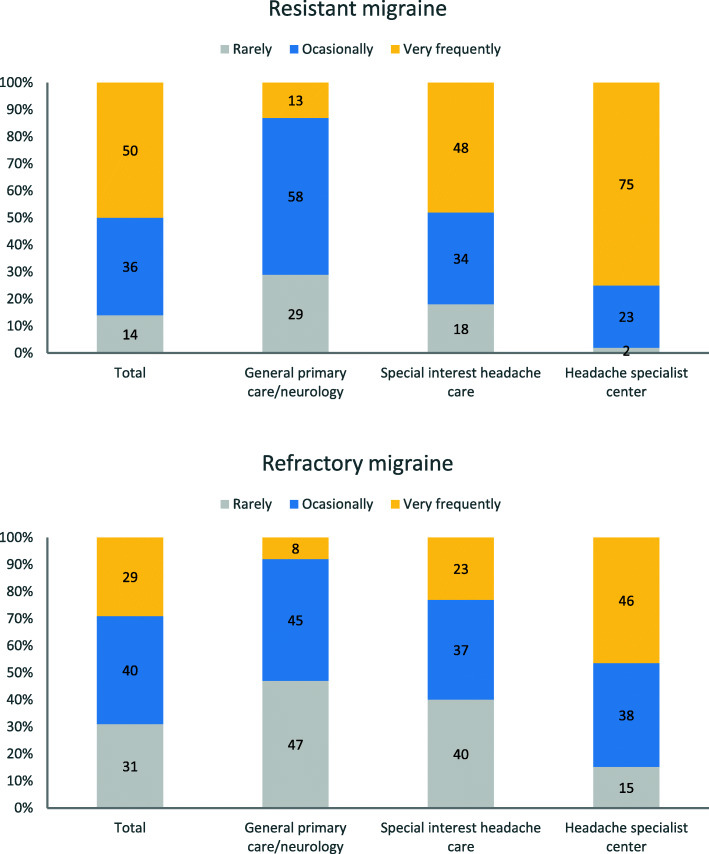
Response to the question “How often do you see in your practice patients with resistant/refractory migraine?”

Resistant migraine was frequently encountered in clinical practice. Overall, 137/277 (49.5%) participants reported that they manage very frequently patients with resistant migraine, 100 (36.1%) participants reported that they manage occasionally those patients, and 40 (14.4%) reported that they manage rarely those patients. Those percentages were significantly different among the three possible levels of care (*P* < 0.0001). In fact, in headache specialist centers, 75% of physicians reported that they manage very frequently patients with resistant migraine; the corresponding proportion was 48% for special interest headache care and 13% for general primary care/neurology (Fig. 2).

Refractory migraine was as well a condition frequently encountered in clinical practice. Overall, 80/277 (28.9%) participants reported that they manage very frequently patients with refractory migraine, 110 (39.7%) reported that they manage occasionally those patients, and 86 (31.0%) reported that they manage rarely those patients. These percentages were significantly different among the different levels of care (*P* < 0.0001). In fact, in headache specialist centers, 46% of physicians reported that they manage very frequently patients with refractory migraine the corresponding proportion was 23% for special interest headache care and 8% for general primary care/neurology (Fig. 2).

**Fig. 3:**
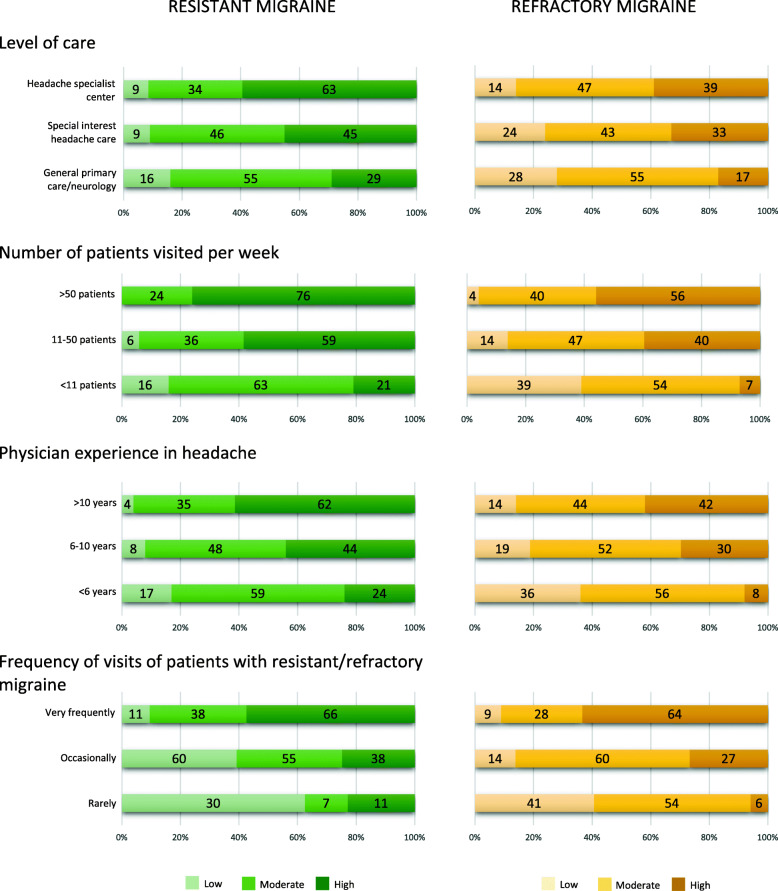
Low, moderate or high confidence in managing resistant and refractory migraine according to characteristics of headache care and physicians

Overall, 22 (8.1%) respondents reported low, 117 (43.3%) moderate, and 131 (48.5%) high confidence in treating resistant migraine, while 57 (20.7%) reported low, 133 (48.4%) moderate, and 85 (30.9%) high confidence in treating refractory migraine As shown in Fig. 3, confidence in treating patients with resistant and refractory migraine was different according to the level of care (*P* < 0.001 and *P* = 0.006, respectively), to the number of migraine patients visited per week (*P* < 0.001 for both), to the years in headache medicine after graduation of the treating physician (*P* < 0.001 for both), and to the frequency of resistant/refractory migraine patients visited (*P* < 0.001 for both).

**Fig. 4:**
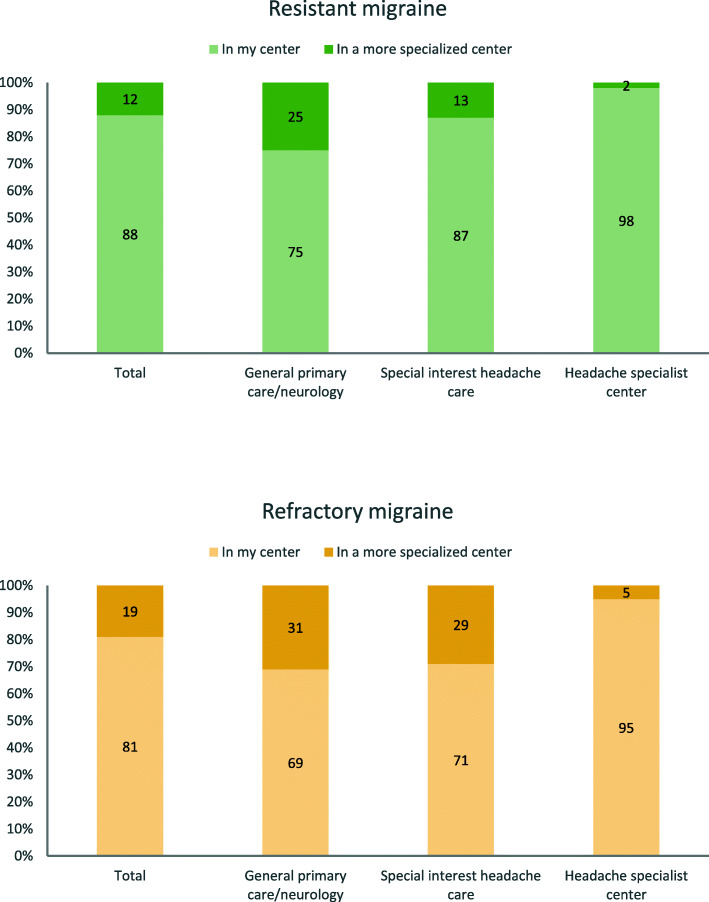
Response to the question “Where are patients with resistant/refractory treated?”

Overall, 245/277 (88.4%) respondents reported that patients with resistant migraine were treated in their own center, while 32 (11.6%) reported that patients were referred to a more specialized center. The corresponding data were 224 (80.9%) and 52 (18.8%) respectively for refractory migraine. Figures were significantly different according to the level of care (*P* < 0.001 for both resistant and refractory), as reported in Fig. 4.

**Fig. 5:**
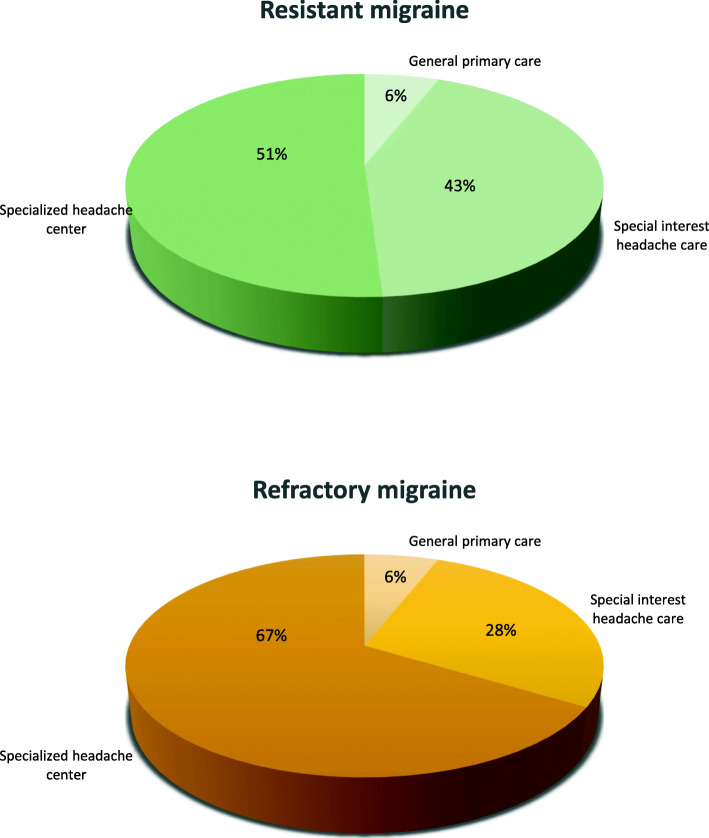
Response to the question “Where should patients with resistant/refractory migraine be managed?”

Referring to the ideal setting of care, 22 (6%) respondents considered that resistant migraine should be managed in general primary care, 162 (43%) in special interest headache care, and 191 (51%) in specialized headache centers. The corresponding figures for refractory migraine were 19 (6%), 95 (28%) and 230 (67%) respectively (Fig. 5).

## Discussion

As new treatment opportunities are entering the migraine filed, new challenges are faced by those who are involved in migraine care and research [15–17]. First, there is the need of reliable criteria, both in clinical practice and in research settings, to select homogenous populations of migraine patients who require treatment escalation. This would ensure a more standardized clinical management of patients and research results’ comparability. Second, while in the past mostly passive acceptance of the lack of successful treatment for some patients with migraine was registered, nowadays researchers are looking for novel migraine specific therapeutic targets. The study of patients who do not have adequate response to the available migraine treatments may provide not only more therapeutic opportunities, but also may improve our understanding in the mechanisms underlying migraine in general.

The present study reports a summary of the impact and attitude toward resistant and refractory migraine. Our data showed that both resistant and refractory migraine are commonly and globally faced in the headache care. Resistant and refractory migraine are particularly common in tertiary level headache centers, where 75% and 46%, respectively, of the physicians use to see very frequently those patients. However, despite being relatively common, many physicians reported a sub-optimal confidence in managing those patients. Remarkably, even in tertiary level headache centers, only 39% of physicians reported high confidence in managing patients with refractory migraine. The figure may be attributed by lack of knowledge of the definition, lack of additional treatment possibilities and lack of guidelines which may help to support those patients. However, physicians with more experience (i.e., more patients visited per week and more years in practice) reported more often a high confidence in treating both resistant and refractory migraine, further highlighting the lack of standardized guidelines and the consequent physicians’ trend to mainly rely on their expertise.

Surprisingly, we found a low referral to more advanced levels of care for patients with resistant and refractory migraine, despite a relevant proportion of physicians operating in the two more basic levels of care expressed from low to moderate confidence in treating those patients. On the other hand, around half of the participants considered that tertiary level headache care is the ideal setting for resistant migraine and 2/3 of participants considered that tertiary level headache care is the ideal setting for refractory migraine.

This preliminary report provides a gross understanding of resistant and refractory migraine, however with some limitations. Responses were based on perceptions and opinions of the participants and bias and inaccuracy. Additionally, generalizability of the findings can be limited. In fact, there were many countries (among them United States and Canada) with lack of responders; the distribution of responders was not homogenous across countries. Due to the limited number of participants per country, we could not perform country-specific analyses. Additionally, also availability of new migraine drugs and the system of care are variable across countries, and this may reflect different attitudes which were not examined by our study. An upcoming filed testing and real-life prospective international study in headache centers across Europe (A real-life study on Resistant and rEFractory migraINE [REFINE]) will provide more insights on the validity of the proposed definitions as well as on the characteristics and clinical course of patients who meet the definition criteria for resistant and refractory migraine.

## Conclusions

Resistant and refractory migraine are conditions which are perceived as common in the clinical practice of those involved in the care of patients with migraine and working in dedicated headache care or centers. Despite being common, there is a lot of uncertainty from physicians, pointing out the need of support. It would be important to set up organized systems for referral of the difficult-to-treat patients from general primary care/neurology and eventually from special interest headache care to tertiary headache centers and to provide guidance on migraine care in situations where the positive clinical response if difficult to achieve. Further research is also needed to clarify the mechanisms which contribute to drug refractoriness in migraine, to understand the role of comorbidities and the therapeutic opportunities arising from combination drugs. Moreover, underlying mechanisms in migraine are far from being entirely clear and there hope that the ongoing future research may shade light on novel targets which may allow the development of new drugs.

## Supplementary Information

**Additional file 1.** EHF survey on resistant/refractory migraine.

## Data Availability

Not applicable.

## Abbreviations

EHF: European Headache Federation
EMHA: European Migraine & Headache Alliance
CGRP: Calcitonin gene-related peptide
mAbs: Monoclonal antibodies
