# Supplementary material for: Burden and attitude to resistant and refractory migraine: a survey from the European Headache Federation with the endorsement of the European Migraine & Headache Alliance
Source: J Headache Pain. 2021 May 18;22(1):39. doi: 10.1186/s10194-021-01252-4 (PMC8130435; doi:10.1186/s10194-021-01252-4)
Supplement: Supplementary file 1 — Additional file 1. EHF survey on resistant/refractory migraine. [file 10194_2021_1252_MOESM1_ESM.pdf]

EHF survey on resistant/refractory migraine

PRELIMINARY AGREEMENT TO PARTICIPATE IN THE PROJECT

The handling of your data will comply with the provisions of the European Parliament and Council Regulation (EU) 2016/679 of 27 April 2016 on Data Protection. All the data will be only diffused in aggregated form.

I declare that I am a physician involved in headache care.	◯ Yes<br>◯ No
I declare that all the information I provide below is adherent to reality.	◯ Yes<br>◯ No
I agree to participate in this project and to be listed among project contributors in all related publications with the affiliation I will report below.	◯ Yes<br>◯ No

PHYSICIAN GENERALITIES AND SETTING

(Those information are mandatory to acknowledge individual participation)

Name	__________________________________
Surname	__________________________________
City	__________________________________
Country	__________________________________
Affiliation (Hospital/University)	__________________________________
E-mail address<br>(We wil use only for future communication related to this project)	__________________________________
Gender	◯ Male<br>◯ Female<br>◯ Other
Years in headache medicine after graduation	◯ ≤ 5<br>◯ 6-10<br>◯ >10
Specialty	◯ Neurology<br>◯ Internal Medicine<br>◯ Primary Care<br>◯ Anesthesiology/Pain<br>◯ Otorhinolaryngology (medicine/surgery)<br>◯ Physical Medicine and Rehabilitation<br>◯ Clinical Pharmacology<br>◯ Obstetrician/Gynecology<br>◯ Other

Which of the following best describes your primary working setting (if you want more details on the definition please have a look at J Headache Pain 2011; 12:419-426: link)?	◯ General primary care/general neurology<br>◯ Special interest headache care (Ambulatory care delivered by physicians with a special interest in headache)<br>◯ Headache specialist center (Advanced multidisciplinary care delivered by headache specialists in hospital-based centers)
Your workingsetting is:	◯ Mainly academic<br>◯ Mainly non-academic
How many patients with migraine (considering both first visits and controls) do you visit per week?	◯ ≤10<br>◯ 11-50<br>◯ >50

Before going ahead with the remaining part of the questionnaire please consider the new definitions of resistan t and refractory migraine by looking at the table below or by dowloading the full paper (find the article)

Resistant migraine	Refractory migraine
**Resistant migraine** can be diagnosed if all the following criteria are met:	**Refractory migraine** can be diagnosed if all the following criteria are met:
A. Established diagnosis of 1.1 Migraine without aura and/or 1.2 Migraine with aura or 1.3 Chronic migraine according to ICHD-III criteria;	A. Established diagnosis of 1.1 Migraine without aura and/or 1.2 Migraine with aura or 1.3 Chronic migraine according to ICHD-III criteria;
B. Debilitating headache for at least 8 days per month for at least 3 months;	B. Debilitating headache for at least 8 days per month for at least 6 months;
C. Failure and/or contraindication to 3 drug classes* with established evidence for migraine prevention, given at an appropriate dose for an appropriate duration.	C. Failure and/or contraindication to all classes with established evidence for migraine prevention, given at an appropriate dose for an appropriate duration.

Specifies to the diagnosis

Definition of debilitating headache

Debilitating headache is defined as headache causing serious impairment to conduct activities of daily living, despite the use of pain-relief drugs with established efficacy at the recommended dose, and taken at the beginning of the attack; failure of at least two different triptans is required.

Drug classes considered for the diagnosis

1. Antidepressant (Amitriptyline, Venlafaxine);
2. Antiepileptic (Topiramate or Valproate);
3. Beta-blocker (Atenol, Metoprolol, Propanolol, Timolol);
4. Calcium channel blocker (Flunarizine or Cinnarizine);
5. Drugs acting on the CGRP pathway (Monoclonal antibodies and Gepants);
6. Angiotensin-converting enzyme inhibitor (Lisinopril) or angiotensin II receptor blocker (Candesartan);
7. OnabotulinumtoxinA (for chronic migraine only);
8. Other pharmacologic preventive treatments with established efficacy in migraine (e.g. any new developed drug).

Definition of drug failure

Failure, at any time, of migraine preventatives because of:

1. lack of efficacy due to persistence of headache with the required frequency for meeting criteria for resistant or refractory migraine, while the patient is on treatment with one of the recognized preventatives at an established dose and for an appropriate duration;§
2. lack of tolerability due to side effects which are unbearable for the patient, cannot be effectively managed and require stopping the drug.

Definition of contraindication

Contraindication is any specific situation in which the use of a given drug is inadvisable.#

Assessment of patients

To validate definitions, we suggest the following:

1. Resistant migraine, evaluation by a Headache specialist with review of medical charts;
2. Refractory migraine, evaluation in a tertiary level Headache Center with in-person follow-up for at least 6 months.

Medication overuse

1. For resistant migraine presence of medication overuse does not exclude the diagnosis;
2. For refractory migraine presence of medication overuse does not exclude the diagnosis but attempts of unsuccessful medication withdrawal needs to be documented.

Additional factors

Triggers and comorbidities need to be considered and managed; unsuccessful management of triggers and comorbidities is possible.

Careful differential diagnosis with mimicking conditions must be done.

PART 2 - RESISTANT MIGRAINE

How often do you see patients who meet criteria for resistant migraine in your clinical practice?	◯ Very frequently (daily/weekly)<br>◯ Occasionally (monthly)<br>◯ Rarely (less than one in a month)
In a scale from 1 (less confident) to 10 (more confident) how do you rate your confidence to treat patients with resistant migraine?	______________________________
According to your opinion, patients with resistant migraine should be managed in (if you want more details on the definition please have a look at J Headache Pain 2011; 12:419-426: link) (multiple choices possible):	☐ General primary care<br>☐ Special interest headache care<br>☐ Specialized headache care
Do you manage yourself patients with resistant migraine or do you refer them to a more specialized center?	◯ I manage them in my center<br>◯ I refer them to a more specialized headache center

PART 3 - REFRACTORY MIGRAINE

How often do you see patients who meet criteria for refractory migraine in your clinical practice?	◯ Very frequently (daily/weekly)<br>◯ Occasionally (monthly)<br>◯ Rarely (less than one in a month)
In a scale from 1 (less confident) to 10 (more confident) how do you rate your confidence to treat patients with refractory migraine?	______________________________
According to your opinion, patients with refractory migraine should be managed in (if you want more details on the definition please have a look at J Headache Pain 2011; 12:419-426: link):	☐ General primary care<br>☐ Special interest headache care<br>☐ Specialized headache care
Do you manage yourself patients with refractory migraine or do you refer them to a more specialized center?	◯ I manage them in my center<br>◯ I refer them to a more specialized headache center
